# Successful Up-Scaled Population Interventions to Reduce Risk Factors for Non-Communicable Disease in Adults: Results from the International Community Interventions for Health (CIH) Project in China, India and Mexico

**DOI:** 10.1371/journal.pone.0120941

**Published:** 2015-04-13

**Authors:** Pamela A. Dyson, Denis Anthony, Brenda Fenton, Denise E. Stevens, Beatriz Champagne, Li-Ming Li, Jun Lv, Jorge Ramírez Hernández, K. R. Thankappan, David R. Matthews, Community Interventions for Health (CIH) collaboration

**Affiliations:** 1 University of Oxford, Oxford Centre for Diabetes, Endocrinology and Metabolism, Oxford, OX3 7LJ, United Kingdom; 2 University of Oxford, Harris Manchester College, Mansfield Road, Oxford, OX1 3DT, United Kingdom; 3 MATRIX Public Health Solutions Inc, 794 Edgewood Avenue, New Haven, Connecticut, 06515, United States of America; 4 InterAmerican Heart Foundation Inc, 7272 Greenville Avenue, Dallas, Texas, 75231-4596, United States of America; 5 School of Public Health, Peking University Health Science Center, 38 Xueyuan Road, Haidian District, Beijing, 100191, China; 6 National Autonomous University of Mexico, Insurgentes Cuicuilco, Coyoacán, 04530, Mexico City, Mexico; 7 Achutha Menon Centre for Health Science Studies, Sree Chitra Tirunal Institute for Medical Sciences and Technology, Trivandrum, India; 8 Oxford NIHR Biomedical Research Centre, Oxford, United Kingdom; Zhejiang University, CHINA

## Abstract

**Background:**

Non-communicable disease (NCD) is increasing rapidly in low and middle-income countries (LMIC), and is associated with tobacco use, unhealthy diet and physical inactivity. There is little evidence for up-scaled interventions at the population level to reduce risk in LMIC.

**Methods:**

The Community Interventions for Health (CIH) program was a population-scale community intervention study with comparator population group undertaken in communities in China, India, and Mexico, each with populations between 150,000-250,000. Culturally appropriate interventions were delivered over 18-24 months. Two independent cross-sectional surveys of a stratified sample of adults aged 18-64 years were conducted at baseline and follow-up.

**Results:**

A total of 6,194 adults completed surveys at baseline, and 6,022 at follow-up. The proportion meeting physical activity recommendations decreased significantly in the control group (C) (44.1 to 30.2%), but not in the intervention group (I) (38.0 to 36.1%), p<0.001. Those eating ≥5 portions of fruit and vegetables daily decreased significantly in C (19.2 to 17.2%), but did not change in I (20.0 to 19.6%,), p=0.013. The proportion adding salt to food was unchanged in C (24.9 to 25.3%) and decreased in I (25.9 to 19.6%), p<0.001. Prevalence of obesity increased in C (8.3 to 11.2%), with no change in I (8.6 to 9.7%,) p=0.092. Concerning tobacco, for men the difference-in-difference analysis showed that the reduction in use was significantly greater in I compared to C (p=0.014)

**Conclusions:**

Up-scaling known health promoting interventions designed to reduce the incidence of NCD in whole communities in LMIC is feasible, and has measurable beneficial outcomes on risk factors for NCD, namely tobacco use, diet, and physical inactivity.

## Introduction

Non-communicable disease (NCD), including cardiovascular disease, cancer, diabetes and chronic respiratory diseases, accounted for over 65.5% of deaths in 2010, with more than 80% of these occurring in low and middle-income countries (LMIC) [1,2]. Diabetes alone caused 5.1 million deaths in 2013 and cost US$548 billion in health spending (11% of the total spent world-wide) in 2013 [3]. Approximately 30% of the deaths in LMIC occur prematurely and are largely preventable [1]. In addition to this premature mortality, NCD is also associated with increased morbidity and reduced quality of life [4], and it has been estimated that the global economic impact of NCD could total US$47 trillion over the next twenty years, equivalent to 5% of GDP [5]. The causes of NCD have their roots in three major modifiable risk factors; tobacco use, physical inactivity and unhealthy diet [6–8]. Prevention of NCD has been moving up the political agenda over the past two years, and initiatives designed to reduce the impact by addressing these modifiable risk factors have now been initiated. Nevertheless, to date there is little evidence for up-scaled interventions to prevent NCD at the population level.

The United Nations (UN) High Level Meeting on NCD in September 2011 included actions that could be taken to reduce NCD risk factors [9], and World Health organisation (WHO) has produced a list of ‘best buys’ in terms of lifestyle change [10], and has recently published a global plan for the prevention and control of NCD [11]. These authorities all recommend evidence-based strategies for lifestyle interventions, but there is limited high-grade evidence for population or community-based approaches and most of the available evidence is derived from studies conducted in high-income countries [12]. Large-scale interventions in communities have been promulgated by some governments, and this is to be encouraged. For example, in the UK, the Change4Life program encourages healthy living [13], but without any systematic evaluation of outcomes.

The traditional medical model of NCD prevention promotes primary prevention—identification and treatment of high-risk individuals—often requiring the use of medication. There is some evidence of efficacy in high-income countries [14], but this strategy may well widen socioeconomic inequalities and is unlikely to translate to LMIC [15]. In addition, primary prevention targets small numbers and largely ignores the community as a whole, and there is little available sign of successful scaling up of prevention programs. For example, there is now strong evidence from randomised, controlled trials of the efficacy of lifestyle interventions to reduce diabetes in high-risk individuals, and yet diabetes prevalence continues to rise around the globe. By contrast, the population approach is inclusive and addresses many factors including health education, structural environmental change, engagement of health providers, transport and education ministries, policy and legislative initiatives and partnerships and coalitions with community organisations. There is evidence from Finland to show that population strategies are effective for reduction in cardiovascular risk and obesity [16–17], and that these effects can be maintained over the long term [18–19]. These population strategies are more effective in reducing risk factors and improving health than the traditional high-risk approach [20], and as a result, the WHO has now called for a paradigm shift to prevention by addressing these different societal factors [11], and the Centers for Disease Control and Prevention (CDC) in the US has recently launched a community strategy designed to combat obesity at the population level [21].

In 2008, the Oxford Health Alliance, a UK registered health charity (No 1117580), began its Community Interventions for Health (CIH) program which was designed to utilise this population approach and which adopted multi-factorial, comprehensive strategies for prevention of NCD by addressing modifiable lifestyle risk factor reduction [22]. CIH is an international collaborative study that took place between 2008–2011 in communities in China, India and Mexico and was designed to reduce the risk of NCD by targeting the three main risk factors of tobacco use, physical inactivity and unhealthy diet. The aim of CIH was to evaluate culturally-specific strategies to (i) decrease the prevalence of smoking and smokeless tobacco use, (ii) improve diet by increasing intake of fruit and vegetables and reducing use of salt and (iii) increase levels of physical activity in local communities in India, China and Mexico.

## Methods

### Study design and participants

The Community Interventions for Health study was designed as a whole community, comparator group study incorporating action-orientated research to examine the prevalence and secular trends of risk factors for NCD. The full methodology for CIH has been reported previously [23]. CIH took place in three different sites in Hangzhou city in China, Kerala in India and in Mexico City. Each country site identified intervention and control areas with a population size between 150,000 and 200,000 people within selected communities and with similar demographic and socioeconomic characteristics. The intervention and control groups were large contiguous areas amenable to intervention, with established community leadership chosen to be appropriately separated to avoid contamination. A community was defined as an administrative unit specific to the country setting e.g. delegacion in Mexico and panchayat in India.

CIH was conducted in four main settings; health centres, workplaces, schools and the community at large. The data reported here relate to information collected from questionnaires administered to adults aged 18–64 years in the community sample. Site-specific sampling frames and random sampling strategies were used at baseline and follow-up to select the sample for evaluation in both intervention and control groups. Questionnaires were administered to a random cross-sectional sample of adults aged 18–64 years using the Kish method to ensure even selection by age and gender [24]. Sampling was undertaken at the smallest administrative unit, and lists of households within those administrative units were accessed and randomly sampled. As needed, new randomized lists were created (without replacement) in order to recruit additional households to reach the required sample sizes for the intervention and control groups. At the household level, the Kish method was used to select individuals.

The study was undertaken according to the Declaration of Helsinki and obtained institutional review board (IRB) approval in each country site (China: IRB00001052-08003 certified by the Institutional Review Board at Peking University Health Sciences Centre, India: IEC/184, Mexico: Oficio JST/1003 /08) and written, informed consent was obtained where required.

### Data collection

As this was a large-scale study with over 750,000 participants, baseline and follow-up data were collected from a stratified, selected sample of adults within each intervention and control site. The information collected included risk factor assessment by means of a questionnaire, which was administered in face-to-face interviews by trained professionals. The questionnaires used for the CIH adult surveys incorporated questions from previously validated surveys including the WHO STEPS [25], the International Physical Activity Questionnnaire (IPAQ) [26], and the Global Adults Tobacco Survey (GATS) [27].

### Interventions

**Fig 1:**
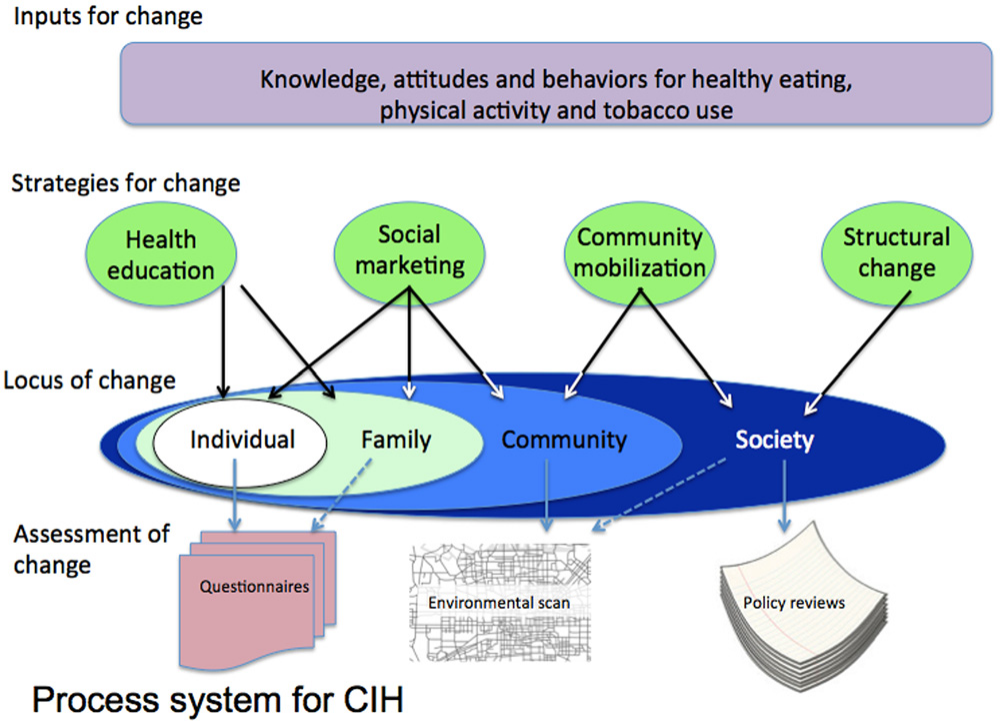
Overview of the process system for CIH.

A menu of evidence-based interventions addressing the three main risk factors was formulated by the CIH international advisory group, and these interventions were summarised in the form of a manual [28]. The intervention strategies used for CIH included structural change, community mobilisation, health education and social marketing and were designed to be delivered in the four settings; neighbourhoods, work places, schools and the community at large (Fig 1). This manuscript reports the results from the main aggregated community sample. Each country site selected culturally appropriate interventions for local application and some examples of these are shown in Table 1. The intervention stage of the CIH project lasted 18–24 months.

**Table 1:** Menu of evidence-based interventions to reduce tobacco use, improve diet and increase physical activity at the community level.

Strategy	Practical applications—examples from CIH
**Tobacco use**	
Promoting smoke-free environments	Encouraging local businesses to ban smoking in the work-place
	Supporting local restaurants to become smoke-free
	Implementing and enforcing smoking restrictions in public areas
Developing counter marketing programmes	Implementing ‘No Tobacco Days’ in workplaces and community centres supported by education about the dangers of tobacco
Providing support groups for tobacco cessation	Working with local health care providers and community groups to set up tobacco cessation groups
Health education and health care	Organising competitions for no smoking posters to be displayed in workplaces, community centres and local recreational areas
	Providing tobacco cessation packs for health professionals to use in clinical practice
	Encouraging health professionals to screen for tobacco use and support smoking cessation
**Diet**	
Encouraging consumption of healthy foods	Increasing affordability by offering subsidies on healthy choices in workplace canteens
	Providing healthy snacks in workplaces
	Increasing accessibility by supporting ‘Grow your own’ schemes and providing vegetable seeds and information
Supporting local farmers markets and communal gardens	Working with local farmers and established markets to provide healthy food to local communities
Promoting institutional policy change	Working with local restaurants, hospital and workplace canteens to add less salt and oil in food preparation, include more fruit and vegetables and to use healthier cooking methods
Providing accurate nutritional information	Displaying nutritional information (energy, salt and dietary fibre) of dishes served in workplace canteens
Using point-of-purchase prompts	Displaying posters in workplace canteens encouraging healthy choices
Health education and health care	Providing salt spoons and oil pots indicating maximum daily amounts to adults in the local community
	Displaying health eating posters in workplaces, community centres and local recreational areas
	Encouraging health professionals to screen and support dietary change
**Physical activity**	
Creating or enhancing access to places for increasing physical activity	Renovating unused public spaces for recreational purposes
	Providing street gyms and fixed exercise equipment in local parks
	Building walking trails along a local canal with stone distance markers
Providing support groups	Introducing sports interest groups in workplaces
	Establishing walking clubs in local communities
Using point-of-decision prompts	Putting posters encouraging stair use near elevators and escalators
	Painting footprints around playgrounds and public recreational areas
Health education and health care	Providing physical fitness testing
	Displaying health eating posters in workplaces, community centres and local recreational areas
	Encouraging health professionals to screen and support increased physical activity
**General risk factors**	
Health education and health care	Distributing health-related messages through the local media outlets including newspapers, local television programmes, bulletin boards and posters
	Building healthy living museums for the general public with self-service health risk evaluation
	Providing public lectures about NCD risk reduction
	Organising health-themed activities around established events such as World Heart Day and World Diabetes Day

### Statistical analyses

The size of the cross-sectional sample for evaluation was based upon predicted small effect sizes (estimated at 6%) between the intervention and control group. The intervention and control groups were assumed to be of equal size, independent of each other and to have similar risk factor prevalence at baseline. Sample size estimation was based upon a two-sided 5% significance test of the null hypothesis that intervention and control groups experience similar changes in prevalence of the three risk factors. Power was fixed at 80% for testing the alternative hypothesis that the intervention group showed a 6% greater change in the key risk factors. The sample size was then arrived at using data of current prevalence of the three risk factors, and the final sample size was selected as the largest across all three risk factors. It was calculated that 2,000 adults in each country site (6,000 adults in total) were needed at baseline and follow-up, comprising a total sample of 12,000 adults.

The study was designed to assess differences in outcomes between the intervention and control group at follow-up to allow for secular trends. Univariate analysis used chi square for nominal variables and Mann Whitney for non normally distributed continuous data. A difference-in-differences analysis (DiD) [29,30] was performed to determine the effect of the intervention. DiD is a version of fixed effects estimation that allows for statistical comparison of the effects of the intervention in the two groups. Comparisons were pre-specified and p = 0.02 was adopted as a conservative significance threshold.

Data were analysed using SPSS v14 (SPSS Inc., Chicago, IL, USA) statistical software package.

### Role of the funding source

None of the major sponsors, namely the National Institute of Health Research, Novo Nordisk A/S and the PepsiCo Foundation had a role in the design or conduct of the study, in the collection, management, interpretation and analysis of the data or in the preparation, review or approval of the manuscript, nor have the data been released to the funding bodies in advance of the publication. The Oxford Health Alliance was responsible for the management and reporting of the study.

## Results

A total of 6,194 adults (48.9% from the intervention group and 51.1% from the control group) completed questionnaires at baseline, and 6,022 adults (50.1% from the intervention group and 49.9% from the control group) completed questionnaires at follow-up. Table 2 shows the characteristics of the sample at baseline.

**Table 2:** Baseline characteristics of adult community sample.

Variable	Control group (C)	Intervention group (I)	Total	p-value
	n = 3164	n = 3030	n = 6194	(I v C)
**Demographics:**				
**Age (years, mean SD)**	40.9 (12.9)	41.5 (13.1)	41.2 (13.0)	0.044
**Gender (%M)**	47.0	47.3	47.1	0.836
**BMI (kg/m** ^**2**^ **, mean SD)**	23.7 (4.4)	24.2 (4.2)	23.9 (4.3)	<0.001
	**%**			
**Risk factors:**				
**Tobacco use:**				
**Male**	35.0	37.2	36.0	0.206
**Female**	4.8	7.3	6.0	0.002
≥**150 mins/week moderate/vigorous physical activity**	44.1	38.0	41.1	<0.001
≥**5 portions fruit and vegetables/day**	19.2	20.0	19.6	0.451
**Salt added in cooking**	90.4	91.7	91.0	0.215
**Salt added at table**	24.9	25.9	25.4	0.357
**BMI** ≥**25 kg/m** ^**2**^	31.5	36.4	33.8	<0.001
**BMI** ≥**30 kg/m** ^**2**^	8.3	8.6	8.5	0.713

### Overweight and obesity

**Fig 2:**
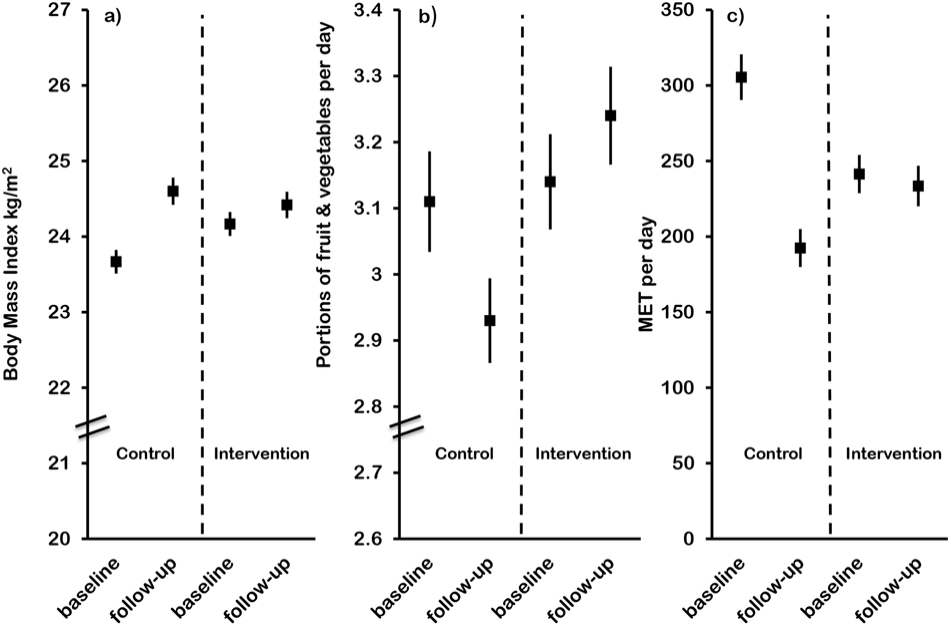
Outcome variables for (a) BMI, (b) portions of fruit and vegetables and (c) physical activity at baseline and follow-up for control and intervention groups.

At baseline, rates of overweight (BMI ≥25kg/m^2^) and obesity (BMI ≥30kg/m^2^) were relatively high, with 33.8% being overweight (and obese) and 8.5% being obese. Fig 2 shows that BMI increased in both groups during the course of the study, but the increase was significantly less in the intervention group compared to the control group with BMI in the control group increased by 0.93 kg/m^2^ compared with a rise of 0.25 kg/m^2^ in the intervention group (p<0.001). Prevalence of overweight and obesity showed a similar trend, with significant increases in both in the control group; overweight increased by 9%, from 31.35 to 40.5% (p<0.001), and obesity by 2.9%, from 8.3% to 11.2% (p<0.001) (Table 3). By contrast, there were no significant increases in either overweight or obesity in the intervention group. However, analysis by DiD showed no difference in the changes between the groups for obesity (p = 0.381), and a suggestion of increased overweight in the control group (p = 0.027), Table 4.

**Table 3:** NCD risk factors at baseline and follow-up in adult community samples.

Risk factor	Control group (C)				Intervention group (I)				I v C at follow-up p-value
	%				%				
	Baseline	Follow-up	Change	p-value	Baseline	Follow-up	Change	p-value	
**Tobacco use:**									
**Male**	35.0	37.7	2.7	0.140	37.2	36.5	-0.7	0.714	0.539
**Female**	4.8	4.6	-0.2	0.834	7.3	5.8	-1.5	0.076	0.116
≥**150 mins/week moderate/vigorous physical activity**	44.1	30.2	-13.9	<0.001	38.0	36.1	-1.9	0.128	<0.001
≥**5 portions fruit and vegetables/day**	19.2	17.2	-2.0	0.037	20.0	19.6	-0.4	0.742	0.013
**Salt added in cooking**	90.4	76.4	-14.0	<0.001	91.7	71.1	-20.6	<0.001	0.001
**Salt added at table**	24.9	25.3	0.4	0.709	25.9	19.6	-6.3	<0.001	<0.001
**BMI** ≥**25 kg/m** ^**2**^	31.5	40.5	9.0	<0.001	36.4	37.9	1.5	0.252	0.076
**BMI** ≥**30 kg/m** ^**2**^	8.3	11.2	2.9	<0.001	8.6	9.7	1.1	0.175	0.092

**Table 4:** DiD odds ratio of risk factors from logistic regression.

Risk factor	Odds ratio	95% Confidence Intervals	p-value
**Tobacco use (male)**	0.730	0.567–0.939	0.014
**Tobacco use (female)**	0.824	0.533–1.274	0.383
≥**150 mins/week moderate/vigorous physical activity**	1.763	1.499–2.073	<0.001
≥**5 portions fruit and vegetables/day**	1.185	0.962–1.459	0.111
**Salt added in cooking**	0.457	0.466–0.900	0.010
**Salt added at the table**	0.605	0.504–0.727	<0.001
**BMI** ≥**25 kg/m** ^**2**^	0.832	0.706–0.979	0.027
**BMI** ≥**30 kg/m** ^**2**^	0.888	0.680–1.159	0.381

### Fruit and vegetable intake

Fruit and vegetable intake was generally low at baseline, with less than 20% eating the recommended five or more portions of fruit and vegetables daily. The generally accepted portion sizes were used: for vegetables this was 85g, or three tablespoons, or a main serving of salad. For fruit this was a medium sized fruit (e.g. apple) or two small fruits (e.g. plum) or a bowl of berries. During the study period, the proportion eating five or more portions of fruit and vegetables decreased significantly in the control group (from 19.2 to 17.2%, p = 0.037), demonstrating an adverse secular trend. By contrast, there was no change in fruit and vegetable intake in the intervention group, showing a significant difference in the secular trend between the two groups at follow-up (Table 3). Mean intakes of fruit and vegetables at baseline were 3.1 portions/day in the control and intervention groups, and at follow-up the control group showed a significant reduction to 2.9 portions/day (p<0.001), with a trend for increase in intake in the intervention group (Fig 2). DiD analysis showed no significant effect of the intervention (p = 0.111), see Table 4.

### Physical activity

Physical activity decreased in the significantly in the control group (from 305 to192 MET/day, p<0.001), with no statistically significant change in the intervention group (Fig 2). Physical activity data was calculated as percentage of the population achieving the recommendation of 150 minutes of moderate or intense physical activity during one week, or at least 30 minutes per day for five days of the week [31]. Less than half of the sample achieved these recommendations at baseline, and at follow-up, this proportion had declined significantly in the control group (p<0.001), again demonstrating an adverse secular trend. There was no change in the intervention group (Table 3). For physical activity, the intervention effect by DiD analysis was significant, p<0.001.

### Tobacco use

The prevalence of tobacco use (smoked and smokeless tobacco combined) was significantly different between men and women at baseline in both the intervention and control groups, the data are therefore reported by gender. Men in the intervention group reduced tobacco use over the course of the study and men in the control group increased tobacco use, although neither change reached significance. However, DiD analysis showed that the reduction in tobacco use was significantly greater in the intervention group compared to the control group (p = 0.014), see Table 4.

In women, there was significantly more tobacco use in the intervention group at baseline, and although both groups reduced tobacco use, there was a trend towards greater reduction in the intervention group.

### Salt intake

A large proportion of the sample added salt in cooking at baseline (91%), although less used salt at the table (25.4%). Both groups showed a significant reduction in the proportion adding salt in cooking at follow-up, p<0.001 for both. The intervention group showed a significant reduction in the proportion adding salt at the table (25.9 to 19.6%, p<0.001) compared with the control group, where there was no significant change in added salt. For both salt added in cooking and at the table, there was a significant difference in the changes in salt between the two groups, see Table 4.

**Fig 3:**
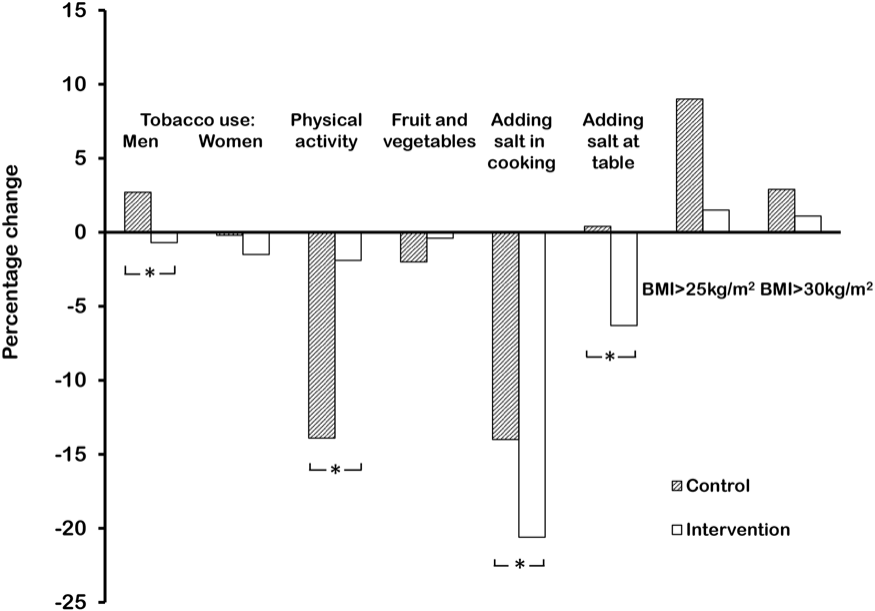
Percentage changes in prevalence of risk factors in control (hatched) and intervention groups. * Significant difference (p<0.02) in changes between control and intervention groups by Difference-in-Difference (DiD) analysis.

Fig 3 compares the changes in prevalence of the risk factors in both groups from baseline to follow-up.

## Discussion

The Community Interventions for Health program has demonstrated that significant reductions in risk factors for NCD can be achieved in targeted populations, and that these types of intervention are suitable for whole communities. In samples from selected areas in China, India and Mexico, interventions to improve health by decreasing tobacco use, increasing fruit and vegetable intake, decreasing salt intake and increasing physical activity have all had some positive impact in intervention areas compared with control areas. The results have shown that this up-scaled approach can influence change in a health-enhancing direction, either by a better positive change or by an impact on the assessed secular trend.

The population approach utilised in CIH, although not a randomised controlled trial, was a comparator study designed to test community interventions and to test the feasibility of scaling up NCD prevention. The interventions used in CIH were multi-component, applied across multiple settings and tailored to the environment with local implementation. They were culturally sensitive and designed and developed locally for each of the different communities and different settings with the aim of replication and sustainability. The China site, for example, introduced a successful public bicycle system to increase physical activity during the course of the study. By contrast, this strategy was unsuitable for India where cycling is dangerous and is seen as a low status activity.

There are important caveats to the interpretation of the study. CIH was designed as a whole community, comparator group study, this being an appropriate and pragmatic method of showing community effects. Engaging all stakeholders in the intervention area was fundamental to achieve the dose effect, and this strategy mitigated against true randomisation. The study design and analysis plan was predicated on the likelihood of small but important possible differences between the two groups at baseline, and this proved to be the case for age, BMI, physical activity, prevalence of overweight and tobacco use in women. The data were analysed by the difference-in-difference methodology to make allowances for secular trends.

In CIH, matching the intervention and control areas for socioeconomic and demographic characteristics meant they were in close geographical proximity, but this increased the likelihood of contamination between the two areas. For example, the city-wide bicycle hire scheme in the China site operated in both control and intervention areas. On the other hand, using distant communities as control areas runs the risks of the demographics of these populations being very different.

In community-based studies, it is challenging to effect significant change at the population level, partly because the dose-effect is so small in this type of study. However, it is important to consider that small changes in large numbers of people can have significant impact on health. Data from blood pressure studies suggest that a reduction as small as 2mmHg in systolic blood pressure is associated with a 10% reduction in stroke mortality and 7% reduction in deaths from ischaemic heart disease [32]. In terms of BMI, an increase of 0.9kg/m^2^ was observed in the control group, with no change in the intervention group. An increase of 1.0kg/m^2^ in BMI is associated with a 25% increase in the risk of type 2 diabetes [33], a 6% increase in the risk of major cardiovascular disease [34] and an 11% increase in the risk of heart failure [35]. Extrapolating these data, it could be speculated that the stability of BMI in the intervention group had benefits compared to the control population in terms of risk reduction in NCD.

The costs of the community interventions were related to the necessity of conducting a trial and the costs of funding the intervention. The majority of CIH costs (80%) were related to trial evaluation. These interventions were undertaken by investigators and stakeholders in diverse cultures, environments and geographies. Widening the interventions to larger communities may well have similar effects, but because of the uncertainties relating to these large-scale interventions, evaluation of outcomes would be wise.

In conclusion, CIH has demonstrated for the first time that wide-ranging, culturally sensitive, community-based interventions for health can be scaled up to a whole population approach, and that this is feasible, affordable and effective in controlling risk factors for non-communicable disease in low and middle-income countries.
